# Hilar mossy cell circuitry controlling dentate granule cell excitability

**DOI:** 10.3389/fncir.2013.00014

**Published:** 2013-02-12

**Authors:** Seiichiro Jinde, Veronika Zsiros, Kazu Nakazawa

**Affiliations:** ^1^Department of Neuropsychiatry, Graduate School of Medicine, The University of TokyoTokyo, Japan; ^2^Unit on Genetics of Cognition and Behavior, National Institute of Mental Health, National Institutes of Health, Department of Health and Human ServicesBethesda, MD, USA

**Keywords:** mossy cells, granule cells, excitability, epileptogenesis, lateral inhibition, hippocampal mossy fibers, pattern separation, temporal lobe epilepsy

## Abstract

Glutamatergic hilar mossy cells of the dentate gyrus can either excite or inhibit distant granule cells, depending on whether their direct excitatory projections to granule cells or their projections to local inhibitory interneurons dominate. However, it remains controversial whether the net effect of mossy cell loss is granule cell excitation or inhibition. Clarifying this controversy has particular relevance to temporal lobe epilepsy, which is marked by dentate granule cell hyperexcitability and extensive loss of dentate hilar mossy cells. Two diametrically opposed hypotheses have been advanced to explain this granule cell hyperexcitability—the “dormant basket cell” and the “irritable mossy cell” hypotheses. The “dormant basket cell” hypothesis proposes that mossy cells normally exert a net inhibitory effect on granule cells and therefore their loss causes dentate granule cell hyperexcitability. The “irritable mossy cell” hypothesis takes the opposite view that mossy cells normally excite granule cells and that the surviving mossy cells in epilepsy increase their activity, causing granule cell excitation. The inability to eliminate mossy cells selectively has made it difficult to test these two opposing hypotheses. To this end, we developed a transgenic toxin-mediated, mossy cell-ablation mouse line. Using these mutants, we demonstrated that the extensive elimination of hilar mossy cells causes granule cell hyperexcitability, although the mossy cell loss observed appeared insufficient to cause clinical epilepsy. In this review, we focus on this topic and also suggest that different interneuron populations may mediate mossy cell-induced translamellar lateral inhibition and intralamellar recurrent inhibition. These unique local circuits in the dentate hilar region may be centrally involved in the functional organization of the dentate gyrus.

## Introduction

The hippocampal formation is critically involved in various brain functions such as spatial memory and navigation (Burgess et al., 2002; Nakazawa et al., 2004), episodic or autobiographical memory (Eichenbaum et al., 1999), and the response to stress (McEwen and Magarinos, 1997). Therefore, any hippocampal impairments potentially lead to cognitive dysfunction or abnormal sensitivity to stress. The hippocampal formation is also often the focus of post-traumatic epileptic seizures, and hippocampal sclerosis is the most common pathology associated with refractory temporal lobe epilepsy (Margerison and Corsellis, 1966). Among hippocampal subregions, the dentate gyrus is the first central information processor, in which granule cells receive sensory inputs from the entorhinal cortex through the perforant path. Granule cell excitation faithfully detonates CA3 pyramidal cells as well as interneurons by mossy fiber axons. Dentate granule cells also send axon collaterals to the dentate hilus (also called the polymorphic cell layer), a region enclosed by the granule cell layer between the upper and lower blades of the dentate gyrus (Amaral et al., 2007). This region has two main classes of neurons, GABAergic interneurons and glutamatergic mossy cells (Soriano and Frotscher, 1994; Wenzel et al., 1997). Importantly, both of these two neuronal cell types are often depleted in the temporal lobe epilepsy patient brains whereas granule cells are less affected. Thus, a long-standing debate has centered on whether hilar neuronal loss is the cause or the consequence of chronic, persistent epileptic activity. In this review, we focus on the function of one type of hilar neuron, mossy cells, and its relation to granule cell activity. In particular, we argue the functional consequence of selective mossy cell loss from our data obtained from an inducible toxin-mediated, mossy cell-ablation mouse line.

## Physiology and connectivity of mossy cells

**Figure 1:**
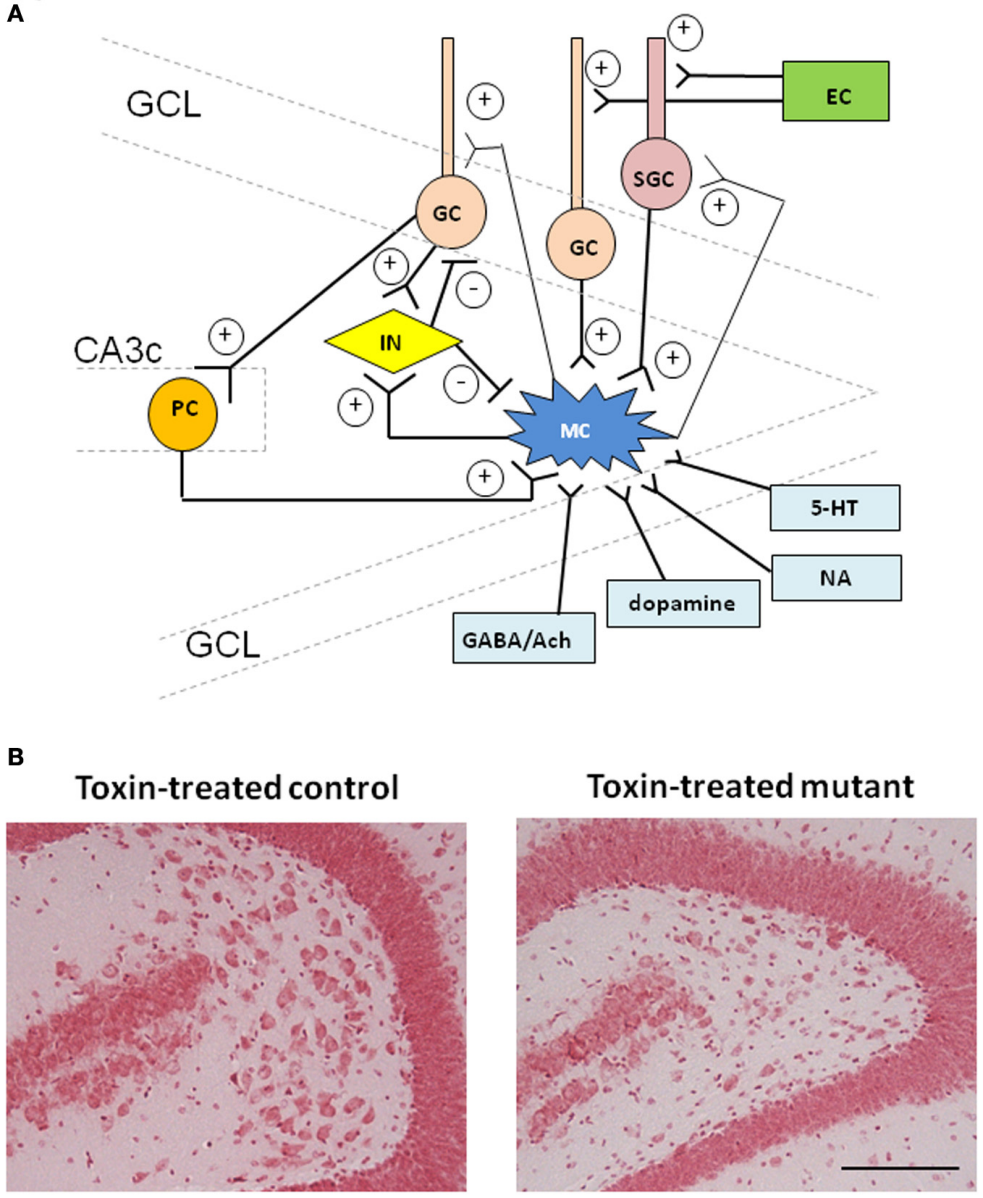
**Schematic of the connectivity of hilar mossy cells and toxin-induced mossy cell degeneration. (A)** Mossy fiber axon collaterals of dentate granule cells are the main input to the mossy cells at their proximal dendrites, called “thorny excrescences.” Mossy cells also receive strong excitatory inputs from semilunar granule cells at the relatively distal dendritic segments of mossy cells. A fraction of CA3 pyramidal cells “backproject” to mossy cells which also receive scarce input directly from the entorhinal cortex. Mossy cells also receive GABAergic inputs from hilar interneurons. Other inputs such as cholinergic and noradrenergic projections are known to modulate mossy cell activity. Mossy cell axons project to the dentate inner molecular layer (IML) along the septo-temporal axis and further contra-lateral hippocampus, where over 90% of asymmetric synaptic contacts are formed on granule cell proximal dendrites as well as semilunar granule cells. Mossy cells also send axon collaterals to dentate GABAergic interneurons in the different lamellae or in the contra-lateral hippocampus. Mutual connections between mossy cells are rare. For simplicity, not all the connections are shown. Ach, acetylcholine; EC, entorhinal cortex; GC, granule cell; GCL, granule cell layer; IN, interneuron; MC, mossy cell; NA, noradrenaline; PC, pyramidal cell; SGC, semilunar granule cell; 5-HT, serotonin. **(B)** Representative photographs of Nissl staining showing histological alterations in the hilar region in CA3c/mossy cell-cre/floxed-diphtheria toxin receptor mutant mouse (right) 4 weeks after diphtheria toxin (DT) administration. Compared to DT-treated control (left), mutant mouse showed the decreased cell number in the dentate hilus. Scale bar, 100 μm.

Glutamatergic hilar mossy cells are known to be highly excitable, because they receive high-frequency large excitatory synaptic potentials (Livsey and Vicini, 1992; Ishizuka and Kosaka, 1998), while they receive 90% less inhibition, as measured by spontaneous inhibitory postsynaptic potentials (IPSPs), when compared with CA3 pyramidal cells (Buckmaster et al., 1993). Other properties, such as their relatively high input resistance together with large anomalous rectifier currents and less spike frequency accommodation, also appear to make them more excitable (Buckmaster et al., 1993). Histological studies have revealed that most of the synaptic inputs to mossy cells arrive via mossy fibers of dentate granule cells (Amaral, 1978; Murakawa and Kosaka, 2001). While there are estimated to be a million granule cells but only ~30,000 mossy cells in rats (Buckmaster and Jongen-Rêlo, 1999), the convergence of mossy fibers onto mossy cells as well as onto CA3 pyramidal cells may be relatively low because granule cells innervate substantially more inhibitory than excitatory cells (Acsády et al., 1998; Mori et al., 2007). Therefore, it is unlikely that the spontaneous activity of mossy cells *in vitro* (Scharfman and Schwartzkroin, 1988; Buckmaster et al., 1992) and *in vivo* (Henze and Buzsáki, 2007) is attributed to the granule cell activity. Alternatively, Williams et al. (2007) recently found that although spiny, granule-like neurons in the inner molecular layer (IML), termed “semilunar granule cells,” project to granule cells, these cells' axon collaterals mono-synaptically excite mossy cells. Since semilunar granule cells receive the input from entorhinal cortex in the molecular layer, it is suggested that semilunar granule cells may provide an alternate pathway for entorhinal inputs to persistently drive hilar neurons and CA3 cells (Larimer and Strowbridge, 2010; Gupta et al., 2012). Interestingly, semilunar granule cells also appear to receive mono-synaptic excitatory input from mossy cells (Williams et al., 2007), potentially making “reverberatory circuits.” As another alternative, mossy cells also receive several other inputs. For example, mossy cells are known to receive excitatory innervation from the CA3 pyramidal cells, which is called “back-projection.” Ishizuka et al. (1990) and later, Li et al. (1994) histologically revealed that CA3 pyramidal cells have collaterals in the hilus, and, particularly, ventral portion of CA3c that was identified as the area with greatest collateralization in the hilus. Additionally, simultaneous recordings in slice preparation showed that immediately after the onset of bicuculline-induced spontaneous bursts in CA3 pyramidal cells, hilar mossy cells, and GABAergic interneurons also demonstrated bursts (Scharfman, 1994a). Because mossy cells send axon to dentate granule cells, these results demonstrated that CA3 pyramidal cells can indirectly activate dentate granule cells via mossy cells under disinhibited condition. A histological study revealed that cholinergic and GABAergic boutons are also abundant around mossy cell somata and on their proximal dendrites, suggesting a direct innervation of hilar mossy cells by GABAergic and cholinergic neurons in the medial septal diagonal band area (Freund and Buzsáki, 1996; Deller et al., 1999). The dentate hilar region also received a prominent noradrenergic input, serotonergic input, dopamine input, and the excitatory inputs from supremammillary area (Amaral et al., 2007) (Figure 1A).

Mossy cells send their associational and commissural axonal projections to the ipsi- and contralateral IML of the dentate gyrus along the extensive longitudinal (septo-temporal) axis (Seress and Ribak, 1984; Amaral and Witter, 1989; Deller et al., 1994; Buckmaster et al., 1996; Wenzel et al., 1997; Zappone and Sloviter, 2001). This raises an important question about how the mossy cells function normally. In 1971, Andersen et al. proposed that the major hippocampal principal cell axons are oriented parallel to each other and course nearly transversally to the long axis of the hippocampus, so that the hippocampal cells are activated in such a near-transverse band, called a lamella, which could represent a functional unit of the hippocampus (Andersen et al., 1971). While this lamella hypothesis was criticized because of a wide, fan-shaped distribution of Schaffer collaterals of CA3 pyramidal cell axons, Sloviter suggested that this idea may be still valid in the dentate gyrus, on the assumption that the net effect of the longitudinal information flow in the dentate gyrus is inhibitory (Sloviter, 1994). Since the mossy fiber projection is known to be longitudinally restricted, his proposal on mossy cell-mediated translamellar lateral inhibition of granule cells was based on the previous anatomical finding in rats that hilar mossy cells longitudinally project to distant lamellae spanning 6–7 mm (Amaral and Witter, 1989). While, alternatively, dentate interneurons receiving mossy cell axons could contribute to translamellar granule cell inhibition, associational projections of any inhibitory interneurons appear to be minimal beyond a longitudinal distance of 1 mm (Struble et al., 1978; Qiu and Han, 1995; Buckmaster and Jongen-Rêlo, 1999; Zappone and Sloviter, 2004; Sloviter and Lømo, 2012).

A central question has been whether translamellar projections of excitatory mossy cells directly excite or inhibit granule cells. Anatomically, over 90% of the axon cloud of mossy cells targets granule cell dendrites at IML more septal or temporal to the lamella where the projecting mossy cell soma is located (Buckmaster et al., 1992, 1996). Furthermore, of these synapses 99% are on dendritic spines belonging to granule cells (Buckmaster et al., 1996; Wenzel et al., 1997). However, the mossy cells also send axons to local interneurons in a translamellar manner, which could inhibit dentate granule cell excitability, thereby contributing to feed-forward inhibition (Misgeld et al., 1992; Scharfman, 1994a; Larimer and Strowbridge, 2008). Supporting this, *in vitro* paired recordings studies by Scharfman have demonstrated that mossy cells mono-synaptically excite both granule cells and interneurons (Scharfman, 1994a, 1995). In addition, she observed polysynaptic inhibition of granule cells in response to mossy cell activity. Therefore, the functional impact of mossy cells has been the subjects of recent controversy (Scharfman and Myers, 2012).

## Mossy cell function

Since mossy cells participate in the recurrent excitatory circuitries with granule cells and also have the potential of inhibiting granule cells via excitation of GABAergic interneurons as described above, it has been hypothesized that mossy cells actively regulate the function of the dentate gyrus (Buckmaster and Schwartzkroin, 1994). Early *in vivo* electrophysiological studies consistently suggested that the excitatory commissural fibers that originate from the mossy cells have a net inhibitory effect on granule cells via activation of inhibitory neurons (Buzsáki and Czéh, 1981; Buzsáki and Eidelberg, 1981, 1982; Douglas et al., 1983; Bilkey and Goddard, 1987). However, it has recently been suggested that there is an excitatory influence of mossy cells on the granule cells under normal conditions (Ratzliff et al., 2004; Myers and Scharfman, 2009). In the following sections, we briefly review the roles that mossy cells are predicted to play in the various dentate gyrus functions, especially memory processing and epileptogenesis.

### Mossy cells in learning and memory

Due to their unique connections, such as feedback excitatory connections to dentate granule cells and back projections from CA3 pyramidal cells, mossy cells have been suggested to play an important role in normal signal processing in learning and memory (Lisman, 1999). Among the dentate functions related to learning and memory, the dentate gyrus actively contributes to pattern separation, defined as the ability to transform a set of similar input patterns into a less similar set of output patterns. An advanced hippocampal computational modeling including mossy cells and hilar interneurons demonstrated that mossy cells provide a mechanism for dynamic regulation of pattern separation (Myers and Scharfman, 2009). This modeling also suggests that pattern separation can be strongly diminished by decreasing mossy cell function and/or by increasing inhibitory hilar interneuron function, while pattern separation can be increased by the opposing manipulations. In support of this model, whole cell recoding of mossy cells *in vitro* by Lysetskiy et al. (2005) predicted that mossy cells can modulate information processing in the fascia dentate. Specifically, their study revealed that, following tetanic stimulation, the mossy fiber synapses on mossy cells showed significant NMDA receptor-independent long-term potentiation, associated with the increased amplitude of EPSCs and decreased failure rates. The mossy fiber synapses on mossy cells also showed activity-dependent short-term plasticity. Interestingly, Hyde and Strowbridge (2012) recently reported in the hippocampal slice preparation that dentate mossy cells reliably encode information as distinct patterns of spontaneous synaptic activity that persist for seconds, and these activities resemble the persistent activity patterns which were previously found in delay periods of working memory tasks. However, overall there are few reports evaluating functional roles of mossy cells in learning and memory, perhaps due to the technical difficulty of manipulating mossy cells *in vivo*.

### Temporal lobe epilepsy and mossy cells

Epilepsy is a neurological disorder characterized by recurrent seizures. There are many different types of epilepsy, which is mainly distinguished by “generalized or partial” and “idiopathic or symptomatic.” Temporal lobe epilepsy, symptomatic partial epilepsy, is the most common type of epilepsy in adults, and is frequently associated with a typical pathological change of hippocampus, called hippocampal sclerosis. One of the pathological characteristics in the tissue of human hippocampal sclerosis is the remarkable neuronal loss in the dentate hilus. It is widely known that hilar cells, mainly glutamatergic mossy cells and GABAergic neuropeptide Y- or somatostatin-positive interneurons, are intrinsically vulnerable to excitotoxic damage (e.g., epilepsy, ischemia, and head trauma), while dentate granule cells appear to be relatively resistant (Sloviter, 1994; Blümcke et al., 1999). Another characteristic consequence of repeated seizures is synaptic reorganization called mossy fiber sprouting, which is an aberrant mossy fiber innervating to postsynaptic targets in abnormal locations, including the granule cell dendrites in the IML (Sutula et al., 1989). Since epilepsy-induced hilar cell loss and mossy fiber sprouting has been replicated in several kinds of animal models, such as pilocarpine-induced or kainic acid-induced epileptic rats (Mello et al., 1993; Buckmaster and Dudek, 1997), the underlying mechanism of hippocampal sclerosis has been extensively investigated for decades using these animal models. However, the exact relation between these pathological changes and epileptogenesis has not been fully understood yet.

While hilar mossy cells are known to be one of the most injury-prone hippocampal neurons, the functional consequence of mossy cell loss has not yet been clarified, regarding whether the net effect is to promote more or less excitability in the dentate gyrus. Therefore, the exact role of mossy cell death in epileptogenesis has long been a matter of debate among researchers. To that end, the following three hypotheses have been proposed and discussed: (1) “mossy cell loss-induced sprouting” hypothesis, (2) “dormant basket cell” hypothesis, and (3) “irritable mossy cell” hypothesis. The first theory, “mossy cell loss-induced sprouting” hypothesis, proposes that the epilepsy-induced loss of mossy cells triggers mossy fiber sprouting, which generates epileptogenesis (Nadler, 2003; Jiao and Nadler, 2007). However, there is no direct evidence that it is specifically the mossy cell loss, as opposed to hilar interneuronal loss, that triggers mossy fiber sprouting. Therefore, it has been questioned that mossy fiber sprouting is epileptogenic.

The second theory, the dormant basket cell hypothesis (Sloviter, 1991), seeks to explain functional consequences of missing excitatory hilar mossy cells. The loss of the mossy cells is thought to deprive interneurons of significant excitatory afferents, leading to a disinhibition of granule cells. In Sloviter's experiment, using the experimental epilepsy model of perforant path stimulation in the rat, an initial 24-h episode of intermittent stimulation-induced hippocampal discharges produced a decrease in the frequency-dependent, presumably feed-forward, inhibition of dentate granule cell discharge, and the development of permanent hyperexcitability in the granule cell populations. These permanent functional changes were replicated in normal rats by a subconvulsive dose of bicuculline, suggesting that the persistent seizure- and damage-associated functional changes may primarily reflect a permanent decrease in GABA_A_ receptor-mediated inhibition of granule cells. In his model, physiological abnormality in dentate granule cell was seen only in animals that exhibited a loss of adjacent dentate hilar mossy cells and hilar somatostatin/neuropeptide Y-inimunoreactive neurons. GABA-immunoreactive dentate basket cells survived despite the extensive loss of adjacent hilar neurons. However, parvalbumin (PV) immunoreactivity, present normally in a subpopulation of GABA-immunoreactive dentate basket cells, was absent on the stimulated side. According to these results, he proposed the following hypothesis. After mossy cell loss, the excitatory synapses on interneurons (e.g., basket cells that inhibit granule cells) in the distant lamella are removed, making these translamellar interneurons hypoactive (“dormant”) and thereby creating a seizure-prone dentate network. On the other hand, mossy cell-driven translamellar interneurons inhibit granule cells under normal condition, thereby dentate information flows only in a particular lamella to CA3 (Sloviter, 1994). Indeed, later Zappone and Sloviter (2004) demonstrated that the focally evoked granule cell activity suppressed distant evoked response ~2.5–4.5 mm longitudinally, and this suppression effect was abolished after extensive hilar cell loss in kainate-treated epileptic rats, suggesting a translamellar lateral inhibition by hilar mossy cells. While this hypothesis triggered much debate, its validity has not been clarified yet (Bernard et al., 1998; Sloviter et al., 2003) because the exact *in vivo* effect of mossy cell-specific loss on dentate network cannot be evaluated by classical pharmacological techniques.

The third theory, named the “irritable mossy cell” hypothesis, proposed that it was not the loss but the survival of mossy cells that played a crucial role in dentate hyperexcitability (Ratzliff et al., 2002). Slice physiology experiment by Santhakumar et al. (2000) reported that, using fluid percussion head trauma model, the percentage decrease in the number of hilar interneurons labeled with either GAD67 or PV mRNA probes following trauma was not different from the decrease in the total population of hilar cells, indicating no preferential survival of interneurons with respect to the mossy cells. Dentate granule cells following trauma showed enhanced action potential discharges, and longer lasting depolarization's, in response to perforant path stimulation, in the presence of the GABA_A_ receptor antagonist bicuculline. Hilar mossy cells in the traumatic dentate gyrus responded with significantly enhanced, prolonged trains of action potential discharges to perforant path stimulation suggesting that surviving mossy cells play a crucial role in the hyperexcitable responses of the posttraumatic dentate gyrus. Regarding the specific effect of mossy cell loss on dentate excitability, their recent experiment revealed that the rapid removal of hilar mossy cells from the dentate network invariably decreased (and not increased) granule cell excitability to perforant-path stimulation (Ratzliff et al., 2004), indicating that the loss of mossy cells in itself is unlikely to directly underlie dentate hyperexcitability. However, in this study, only a small subset (roughly 5–20%) of mossy cells were acutely ablated in slice preparation, which might have been insufficient to see some of the effect described by Zappone and Sloviter (2004).

The reason why little is known about mossy cells function could be due to the lack of specific marker which is commonly available between species, especially rat and mouse. Another reason for the difficulty could be that, unlike other principal excitatory cell types of the hippocampus, mossy cells do not form recognizable layers, and are scattered in the hilar region, which makes their *in vivo* accessibility for physiological studies difficult (Henze and Buzsáki, 2007). Therefore, to solve this question, an animal model in which mossy cells can be selectively manipulated, such as mossy cell-specific genetically-engineered mouse, has long been required.

## Mossy cell-specific ablation mice

Recently we have generated a transgenic toxin-mediated, mossy cell-degeneration mouse line, by crossing the mossy cell/CA3-restricted Cre line with forebrain-restricted *loxP*-flanked diphtheria toxin receptor (fDTR) line (Jinde et al., 2012). Upon *i.p.* injection of diphtheria toxin (DT), immunostaining of GluA2/3 and calretinin (mossy cell markers) and Fluoro-Jade B staining for labeling neurodegeneration revealed that this mossy cell-restricted diphtheria toxin receptor expression line (mossy cell-DTR, hereafter referred to as mutant) showed an extensive mossy cell degeneration. The degree of degeneration was up to nearly 80% within a week and eventually 90% after 1 month, throughout the longitudinal axis of both dorsal and ventral hippocampus (Figure 1B). Whereas degeneration was also observed sparsely in area CA3c, subregion of area CA3 which is located close to the granule cell layer, there was no statistical difference in the cell number of CA3c between before and after DT administration and no other brain areas including dentate granule cells were affected. In contrast, DT-treated control mice, regardless of genotypes, showed no neurodegeneration. Therefore, using this mutant mouse, the impact of mossy cell-specific neuronal degeneration on dentate physiology and behavior can be evaluated. The major findings following toxin-induced mossy cell degeneration are summarized in Table 1.

**Table 1:** **Summary findings after mossy cell-selective neurodegeneration**.

	**Acute phase (4–11 days)**	**Chronic phase (~6 weeks)**	**Comments**
**Morphology**	No obvious change except mossy cell degeneration	No mossy fiber sprouting; GABAergic sprouting at IML	Mossy cell loss triggers no mossy fiber sprouting
**sEPSC and sIPSC from granule cell**	Frequencies both reduced	Back to normal level	Transient reduction of both excitatory and inhibitory input to granule cell
***In vitro* perforant path stimulation**	Hyperexcitable granule cell	Back to normal level	Net inhibitory role of mossy cell in granule cell activity
**Kainate-induced IEG expression**	Increase in c-Fos and Zif268	No change	Associated with hyper-excitable granule cell
**Kainate-induced seizure**	More susceptible	No change	Possibly due to hyper-excitable granule cell
***In vivo* LFP recording**	No epileptiform discharge	No epileptiform discharge	Mossy cell loss insufficient for spontaneous epilepsy
	Theta power enhanced during exploration	Back to normal level	Possibly due to hyper-excitable granule cell
**Behavior**	No behavioral seizure	No behavioral seizure	Mossy cell loss insufficient for spontaneous seizure
	Increased anxiety	Back to normal level	Possibly due to hyper-excitable granule cell
	Impaired contextual discrimination	Back to normal level	Possibly due to hyper-excitable granule cell

IEG, immediate early gene; IML, inner molecular layer; LFP, local field potential; sEPSC, spontaneous excitatory postsynaptic current; sIPSC, spontaneous inhibitory postsynaptic current.

### Evaluation of epileptogenesis theories regarding mossy cell function

Our findings have several implications for the existing theories regarding the role of mossy cell loss in epileptogenesis. First, our results revealed that acute granule cell hyperexcitability follows the selective and extensive degeneration of mossy cells, which provides the first direct evidence for a net inhibitory role of mossy cells in the dentate gyrus. Thus, these results support the central tenet of the “dormant basket cell” hypothesis, which states that mossy cell loss reduces the excitatory drive onto the inhibitory basket cells, hence they become “dormant” and as a result, dentate granule cells become hyperexcitable (Sloviter, 1991; Sloviter et al., 2003). The “dormant basket cell” hypothesis postulates that reduction in GABAergic inhibition results in seizures, and this is consistent with the diminished GABAergic inhibition observed in models of temporal lobe epilepsy and during status epilepticus.

Second, despite of strong support for dormant basket cell hypothesis, our findings also indicate that the granule cell hyperexcitability caused by mossy cell loss in this mouse model is apparently not enough to cause spontaneous granule cell epileptiform discharges or spontaneous behavioral seizures. Granule cells are possibly powerful amplifiers of excitation, but the disinhibited granule cells alone may not generate spontaneous epileptiform discharges unless abnormal excitatory inputs elicit spontaneous epileptiform discharges in granule cells. One plausible explanation is the involvement of the entorhinal cortex and other related structures in temporal lobe epileptogenesis. Schwarcz and colleagues (Du et al., 1993) suggested that selective neuronal loss in the entorhinal cortex plays a pathophysiological role in epileptogenesis. Indeed, in perforant path-stimulated rats, spontaneous granule cell epileptiform discharges began immediately after stimulation, and preceded spontaneous behavioral seizures (Bumanglag and Sloviter, 2008). It is conceivable that generation of spontaneous epileptiform discharges requires aberrant excitatory input from the entorhinal cortex onto disinhibited dentate granule cells.

Third, we did not see any signature for “irritable” mossy cells, even if 10–20% of mossy cells survived in the mutants, because sEPSC frequency of granule cells in mutant mice was robustly reduced upon mossy cell degeneration. Accordingly, granule cell hyperexcitability in the mutants is unlikely due to the hyperexcitability of surviving mossy cells after DT treatment. However, we do not exclude a possibility that surviving mossy cells play a role in spreading excitability through their long-range direct connections to granule cells.

Fourth, our findings suggest that mossy cell loss alone is insufficient to trigger mossy fiber sprouting. As one of potential mechanisms of reverberating excitation in the dentate gyrus of epileptic brains, mossy fiber sprouting has been extensively studied since the original findings of Nadler and colleagues (Tauck and Nadler, 1985). However, it remains elusive as to what causes mossy fiber sprouting. One idea was that the sprouting is driven by the degeneration of and/or loss of innervation from mossy cells, because in the pilocarpine-induced status epilepticus rats, the extent of mossy fiber sprouting is correlated with the number of mossy cell loss (Jiao and Nadler, 2007). Contrary to this assumption, we did not observe any Timm staining-positive immunoreactivity in the IML even 6–8 weeks after DT treatment.

Finally, instead of mossy fiber sprouting, we observed compensatory GABAergic sprouting onto IML which began as early as 2 weeks after DT treatment and gradually progressed until 6–8 weeks after mossy cell degeneration. Concomitantly, the sIPSC frequency from the mutant granule cells, which was transiently decreased during the acute phase, returned back to the normal level by the chronic phase. This suggests that this slow process of synaptic reorganization may reverse the acute granule cell hyperexcitability.

### Evaluation of mnemonic theory regarding mossy cell function

Our findings may also shed light on the mechanisms of dentate pattern separation. There are several possibilities that may explain the context discrimination deficits observed following mossy cell loss in the mutant mice. First, electrophysiological recordings from dentate gyrus of awake behaving rats demonstrated decorrelated firing patterns of the dentate granule cells in response to subtle changes in input as evidence for pattern separation (Leutgeb et al., 2007). Therefore, it is reasonable to assume that following mossy cell loss in the mutants the overall excitability increase in granule cells disrupts their ability to fire dissimilar to each other in the context discrimination tasks. A similar disruption may be elicited by cell-type specific ablation of functional NMDA receptors in the granule cells (McHugh et al., 2007).

Alternatively, mossy cell loss may disturb the feed-back regulation by CA3 cells of the pattern separation function, since mossy cells appear to be under direct modulation by CA3 axons, that back-project to the dentate gyrus (Scharfman, 2007). However, there must be some mechanisms that compensate for this potential disturbance by the chronic phase in the mutants, because contextual discrimination deficits were no longer observed in the chronic phase.

Finally, considering a likely role of newborn granule cells in the mnemonic processes (Aimone et al., 2011; Sahay et al., 2011), it is plausible that mossy cell loss may impair dentate neurogenesis, thereby disrupting pattern separation. However, in this mouse model, contextual discrimination deficits are detected 1 week after toxin treatment, but not during the chronic phase when any possible effect of mossy cell loss in the neurogenesis should be seen. We also observed no detectable impact of mossy cell loss on adult neurogenesis as assessed during the chronic phase (Jinde et al., 2012). Further studies are necessary to delineate the pattern separation deficits in this mutant.

### Limitation of this mouse model

There are a few limitations of the mossy cell deletion mouse model (Jinde et al., 2012) that need to be acknowledged and addressed. The first limitation is the extent to which the mossy cells were not quickly ablated. We found that toxin-treatment induces selective mossy cell degeneration up to over 75% of cells within a week and over 90% after 1 month throughout the longitudinal axis of both dorsal (septal) and ventral (temporal) hippocampus. This is quite extensive; however, it is still possible that the 10–20% of surviving mossy cells may play a role in epileptogenesis and pattern separation through their long-range direct connections to translamellar granule cells. Indeed, the longitudinal connections were cut in our horizontal slice preparation from ventral hippocampus in our study (Jinde et al., 2012). It is also noted that continuous EEG recordings across many days is required to detect any subtle epileptiform activity. There are also concerns about the mouse strain which we used. Our transgenic mouse model is in the C57BL/6 strain background. Systemic injection of pilocarpine results in robust mossy fiber sprouting in the C57BL/6 mice that survived status epilepticus (Shibley and Smith, 2002). However, after status epilepticus induced by kainic acid injection, the C57BL/6 strain of mice is also known to be more resistant to hilar cell loss and to mossy fiber sprouting as compared to other mouse strains (Schauwecker and Steward, 1997; Schauwecker et al., 2000; McKhann et al., 2003). The use of C57BL/6 strain in our study may reduce the vulnerability to spontaneous seizure, and may make these mice more resistant to mossy fiber sprouting. It will be interesting to genetically ablate the mossy cells in more seizure-prone strain background for seizure assessment and sprouting analysis.

### Translamellar lateral inhibition by basket cell-like interneurons

Our study using mossy cell degeneration mutant mice revealed that mossy cell-driven GABAergic interneurons play a crucial role in granule cell inhibition. Since mossy cells presumably project to virtually all types of interneurons across lamellae, except interneuron-selective cells (Zappone and Sloviter, 2004; Dyhrfjeld-Johnsen et al., 2007), most interneuron types could contribute to the granule cell lateral inhibition. Are there any specific types of interneurons involved in this inhibition? Spontaneous IPSC events from granule cells of mutants and controls in the presence and absence of glutamate receptor antagonists (NBQX and D-AP5) suggests that there are at least two sources of inhibition that can be found in the granule cells in the horizontal cut slices of dentate gyrus: 30% may originate from interneurons that receive intra-lamellar input from glutamatergic mossy cells, while the remaining (70%) may receive no contribution from mossy cells in slice preparation. Since sIPSC frequencies are nearly the same between control slice with the blockers and the mutants, we expect that only mossy cells, but not any other glutamatergic excitatory neurons, are driving interneurons in the same lamella in our slice preparation, thereby increasing the sIPSC events by 30% in the control mice.

**Figure 2:**
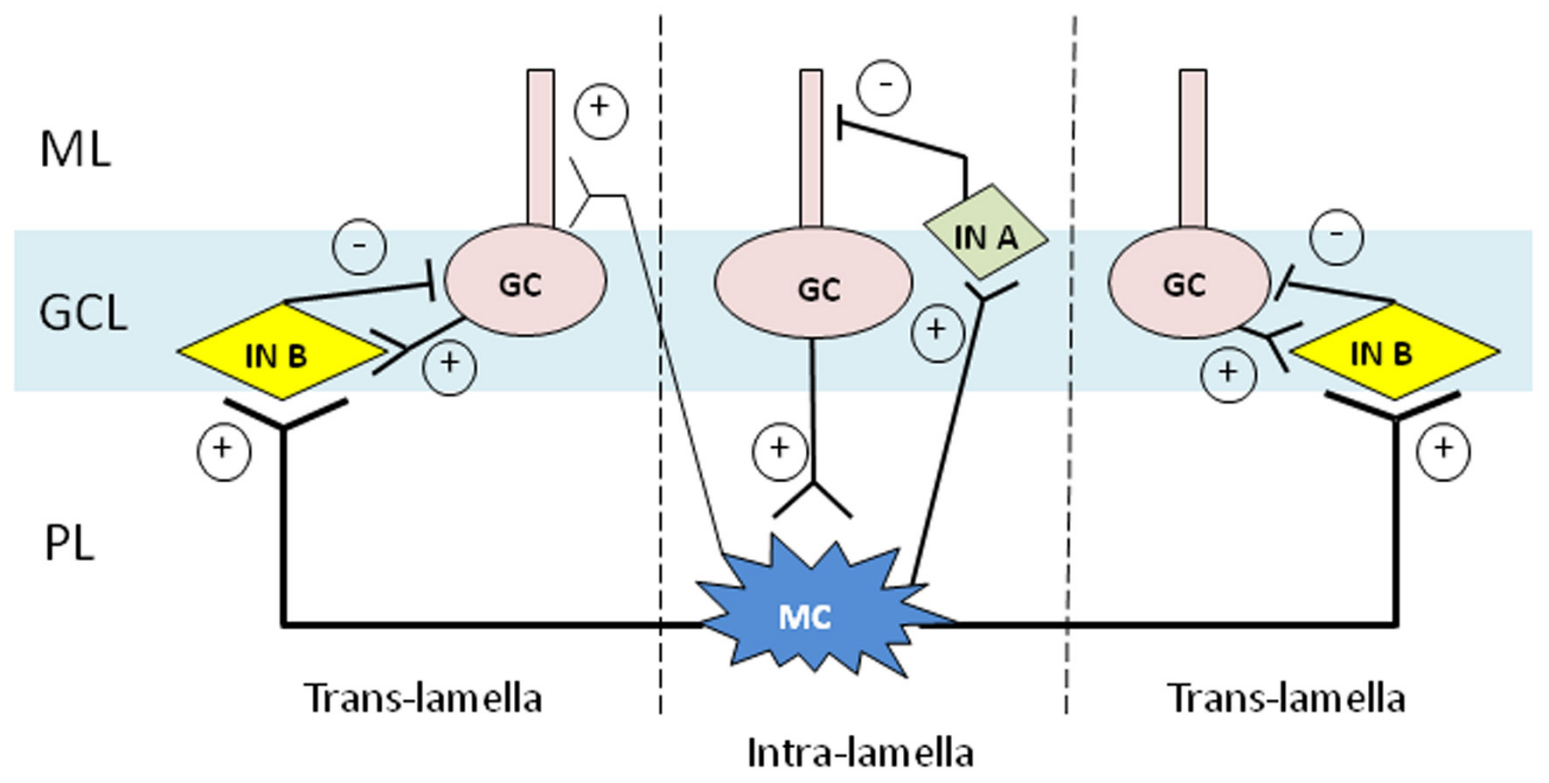
**Two hypothetical modes for mossy cell-driven feed-forward inhibition of granule cells.** Based on our findings, dentate granule cells appear to be inhibited by two distinct categories of interneurons in light of fast- or slow-rise kinetics of postsynaptic GABA_A_ receptors. We propose that granule cells located in the same lamellae receive inhibition from interneurons (In A; interneuron A) which display slow-rise time kinetics of sIPSCs at the granule cell dendrites. Conversely, granule cells translamellar to the mossy cells may receive perisomatic inhibition from interneurons (In B; interneuron B) that display fast-rise time kinetics. While the nature of those interneurons is uncertain, we suspect interneurons translamellar to the mossy cells are basket cell-like cells. For simplicity, the dendrites of mossy cells and interneurons are omitted. GC, granule cell; GCL, granule cell layer; MC, mossy cell; ML, molecular layer; PL, polymorphic layer.

Furthermore, analysis of granule cell-rise time separated in these preparation raises a possibility that two distinct types of interneurons that provide IPSCs of, project to the same granule cells, in relation to the location of mossy cells activating them (Figure 2). One group of interneurons target granule cells with IPSCs of slow-rise time kinetics, and they are located close to the mossy cells, e.g., they receive excitation from those mossy cells in an intra-lamellar manner. Another group is the interneurons with fast-rise time kinetics, which are located translamellar to the mossy cells, again receiving excitatory projection from those mossy cells. Since slow-rise time kinetics interneurons compose of only 30% of inhibition, we think mossy cell feed-forward inhibition is largely translamellar, which was first demonstrated by Zappone and Sloviter (2004).

It is widely accepted that properties of the presynaptic cells as well as the target cells determine the synaptic properties (Klausberger and Somogyi, 2008). Interestingly, there is some emerging evidence that GABA_A_ receptor subunits inserted at the synapse also depend on the types of presynaptic interneurons. Kinetics of GABAergic transmission depends on the subunit composition of the GABA_A_ receptors, and α subunits are particularly important among others. For example, GABAergic transmission involves α1 subunit at fast-spiking basket cell to CA1 pyramidal cell synapses (Thomson et al., 2000), and α2/3 subunits are expressed at much higher density at PV-negative basket cells synapses, and α2 or α2/3 often found in the receptors on the axon initial segments, while less present in somatic and dendritic synapses (Nusser et al., 1996; Loup et al., 1998; Pawelzik et al., 1999; Nyíri et al., 2001). Synapses with α1, α2, or α3 subunit containing GABA_A_ receptors usually demonstrate fast-rise time kinetics, while α5 GABA_A_ receptor subunits which are utilized for example, at the bitufted cell to pyramidal cell synapses in the somatosensory cortex display slower rise time kinetics (Ali and Thomson, 2008).

According to anatomical studies, we know that most interneuron types in the dentate gyrus receive inputs from mossy cells and that mossy cells are involved in translamellar lateral inhibition (Buckmaster et al., 1996; Zappone and Sloviter, 2004). Therefore, based on our results, we predict that a majority of translamellar lateral inhibition to granule cells with fast kinetics is derived from basket cell-type interneurons that display fast-rise time kinetics. In other words, mossy cells may preferentially project to basket cell-like interneurons in a translamellar manner, which exert a powerful synaptic inhibition onto granule cells. Therefore, it is plausible that hilar mossy cell loss results in a robust lateral or translamellar disinhibition of granule cells by the “dormant basket cells,” which may contribute to the epileptogenesis after post-traumatic injury or prolonged febrile seizures (Sloviter, 1994). The types of interneurons with slow-rise time kinetics which are located in an intra-lamellar manner to mossy cells remain to be clarified. However, our finding that IPSC fast-rise time from mutant granule cells is somehow similar to that of the control granule cells after glutamate receptor blockade suggests that glutamatergic mossy fiber projection to those interneurons has minimal impact on granule cell inhibition. It is possible that dentate molecular layer interneurons (so-called MOPP cells) play a role in intralamellar inhibition of granule cells because mossy fibers do not project to these MOPP cells (Halasy and Somogyi, 1993). Alternatively, mossy fiber input to the dentate interneurons may be negligible in slice preparation because the granule cells do not fire spontaneously. In this case, any types of interneurons, including HIPP (Hippocampal Interneurons of the Perforant Path) cells, may serve as type B interneuron (“In A” in Figure 2) in our model. Future study is necessary to assess our hypothesis presented here.

## Summary and conclusions

Here we presented an overview the characteristics of mossy cells, including connectivity, physiology and function. We described the involvement of mossy cells in temporal lobe epilepsy and mnemonic processes, reviewing several hypotheses addressing the effect of mossy cell loss. We further summarized the recent results from our transgenic mouse that exerts toxin-mediated mossy cell degeneration. The toxin-induced selective mossy cell degeneration resulted in acute granule cell hyperexcitability, which suggests a net inhibitory effect of mossy cells. Despite this, no epileptic seizure or mossy fiber sprouting was observed after mossy cell degeneration. Based on our *in vitro* slice recording using the mutant mice, we hypothesize that there are two distinct populations of mossy cell-driven interneurons which inhibit dentate granule cells. One is a type of interneurons with slow-rise time kinetics of sIPSCs at the granule cell synapse which receive inputs from mossy cells in an intra-lamellar manner. Another one is a basket cell-like interneuron-type with fast-rise time kinetics which receives excitatory input from mossy cells in a translamellar manner. Since our study as well as previous literature (Zappone and Sloviter, 2004) demonstrated that the translamellar lateral inhibition seems to be dominant, it is hypothesized that a majority of mossy cell-mediated granule cell inhibition is derived from basket cell-type interneurons with fast-rise time kinetics driven by mossy cells in a translamellar manner. These results may contribute to clarifying the underlying mechanism of epileptogenesis and the dentate function which is involved in learning and memory.

### Conflict of interest statement

The authors declare that the research was conducted in the absence of any commercial or financial relationships that could be construed as a potential conflict of interest.
